# Pattern and Predictors of Unmet Supportive Care Needs in Cancer Patients

**DOI:** 10.7759/cureus.1234

**Published:** 2017-05-09

**Authors:** Paul T Okediji, Omolola Salako, Olamijulo O Fatiregun

**Affiliations:** 1 Research & Development, Sebeccly Cancer Center, Lagos; 2 Department of Radiotherapy, Lagos University Teaching Hospital; 3 Department of Psychiatry, Lagos University Teaching Hospital

**Keywords:** unmet needs, perceived needs, supportive care needs, needs assessment, cancer, malignancy, quality of life

## Abstract

The incidence of cancers is increasing and this is associated with an increase in the burden of the disease. Patients with cancer have to deal with reduced physical functioning, emotional instability, difficulty in concentrating, and an overall diminished feeling of well-being. This creates deficits that have not been well catered for by traditional cancer care, leading to an overall dissatisfaction with care and a reduced quality of life. This review aims at assessing the pattern of unmet needs in cancer patients and to provide information as to the factors that influence the perception of unmet needs. Studies directly focused on unmet needs in cancer patients which were retrieved from Medical Literature Analysis and Retrieval System Online (MEDLINE), Public/Publisher Medline (PubMed), PsychINFO, Excerpta Medica database (EMBASE), and Google Scholar; from the earliest records till 2016. Unmet needs in cancer patients have been measured with a wide variety of tools, with the supportive care needs survey (SCNS) being the most commonly used as a result of its strong psychometric properties, ease of use, responsiveness, and its coverage of the major domains of unmet needs. The most common unmet needs were in the domains of health system and information, psychological, and physical and daily living. These needs are influenced by sociodemographic factors such as age, sex, marital status, income level; and clinical factors such as location of cancer, stage of disease, and tumor size. It is clear that cancer patients experience a wide range of unmet supportive needs, for which solutions need to be devised in order to improve the supportive care services for these patients and their overall quality of life.

## Introduction and background

### Introduction

In spite of recent advances in scientific knowledge on the pathogenesis of cancers, the incidence and burden of cancers are still on the increase globally. Cancer has been reported as being the second most common cause of mortality in today’s world and governments have recognized it as a major threat to national health [1]. The GLOBOCAN report of 2012 shows that there were over 14 million new cases of cancer and 8.2 million deaths from cancer in 2012 [2]. In the United States alone, more than one million new incidences of cancer occur every year and this contributes about one-fifth of all deaths in the country [3]. About 273,000 new cases of cancer are reported in the United Kingdom and more than half die every year from the disease. The most commonly diagnosed cancers are breast, prostate, colorectal and lung cancers. The increasing prevalence of cancers have been attributed to increasing life spans, improved health indices, increased awareness, early detection, and improved cancer care; all of which make cancer patients live longer [1, 3-4]. However, with this increasing prevalence comes an increased burden, especially in terms of morbidity.

The burden of cancer will be expressed in terms of morbidity in this review, and this has been estimated using a number of parameters such as the quality of life, patients’ satisfaction with care and now, an assessment of the needs of the patients. Over the years, quality of life studies have indicated that cancer patients have to deal with a broad variety of problems ranging from the inability to cope with social relationships, difficulties with home and family management; sleep challenges, impaired work productivity, and sexual dysfunction [3, 5]. More recently, the concept of unmet needs has explained the relationship between cancer diagnoses and these problems.

Evidence have shown that a diagnosis of cancer places a significant psychological burden on the patients. Many patients experience a temporary decline in their quality of life immediately after a cancer diagnosis [6]. The diagnosis also forces patients to alter their perspectives on their health and even life itself [7]. Fong and Cheah [8] report that many of these patients are weighed down by the inability to concentrate, emotional instability, issues of physical lethargy, reduced physical functioning and an overall diminished feeling of well-being. Asides the diagnosis, the management of the disease itself leads to significant struggles with the side effects of therapy, physical complications of the disease, psychological sequelae such as anxiety, uncertainty, hopelessness, depression, fear of recurrence, and impaired body image; and social challenges such as sexual dysfunction, difficulty in maintaining social relationships, marital distress, and impairment in the usual day-to-day functioning [9-10]. Unfortunately, it has been found out that many cancer patients do not receive enough support or care to adjust and cope with these challenges [10-11]. This has led to a growing interest in this field and the focus on the concept of unmet needs.

The term “unmet needs” loosely denotes the deficiencies in every area of the patients’ life that arise as a result of having to deal with a diagnosis of cancer or any other chronic ailment. These needs can develop at any point in the disease trajectory, from the point of detection and diagnosis to the completion of treatment, or death, as the case may be. Unmet needs have been categorized into these major domains: informational, emotional, psychosocial, practical, spiritual, and physical [12]. Research has suggested that the largest number of unmet needs is in the psychological domain [10, 13-14]. The next common area of need is in health information [10, 13, 15]. Other areas of need are found in the domains of physical and daily living, sexuality, and patient care, to a much lesser frequency [9, 16].

Asides the influence of medications and other forms of therapy on the effectiveness of cancer care, the magnitude of unmet needs may negatively influence the outcome of cancer therapy. Studies have shown that unmet supportive care needs lead to ineffective coping, worsened emotional distress and a reduced quality of life [17-18]. The relationship between unmet needs and quality of life has been the focus of a few studies. Park and Hwang [19] show that unmet psychological, sexual and physical needs are strong predictors of the quality of life of cancer patients and that the influence of unmet needs on the quality of life of breast cancer patients is more significant than the effect of clinical or socio-demographic characteristics. Similarly, many other authors have argued that the more the number of unmet needs across all the domains of need, the lesser the quality of life of the patient.

It is apparent that studies have identified the relationship between unmet supportive care needs and quality of life. However, they fail to indicate which of these needs, patients feel, they require support and help with the most. The assessment of needs identifies and evaluate the specific issues that patients experience and the perceived magnitude of these issues. Since need denotes a necessary resource or required action that is desirable or important for the attainment of optimal well-being, a proper assessment of needs makes it possible for patients with significant levels of need to be easily identified and targeted with the required interventions [3]. It also allows for the prioritization and focused improvement of specific aspects of health services [20].

Studies have established the high prevalence of unmet needs amongst cancer patients. However, there are variations in the magnitude of unmet needs experienced by the patients based on the type of cancer they suffer from. Patients with breast cancer or melanoma and those with mixed cancers have been reported to have higher levels of unmet needs in the domain of health information provision, while patients with other types of cancers have more unmet needs in the physical/daily living needs domain [3]. This suggests the influence of factors such as the type of cancer, stage of the disease, and type of assessment instrument used. In addition, the influence of specific sociodemographic factors such as age, gender, marital status, income level, occupation, family size, and a number of children on the magnitude and distribution of unmet have been highlighted [3- 4, 6, 8, 10, 19]. In order to prognosticate and predict unmet needs in newly diagnosed cancer patients, it is important to fully examine and understand these factors that influence the perception of unmet needs and other methodologic limitations involved in the assessment of unmet needs. This is the purpose of this review, to give an in-depth understanding into the prevalence and possible predictors of unmet needs through a review of previous research.

### Methods

This review was carried out to critically evaluate primary research that had been done on the supportive care needs of patients with cancer. The authors searched online databases for peer-reviewed journal articles on MEDLINE, PubMed, PsychINFO, EMBASE, and Google Scholar from the earliest records until 2016. Titles and abstracts of relevant studies were searched for with various combinations of the following keywords: unmet needs, perceived needs, supportive care needs, needs assessment, cancer, breast cancer, lung cancer, prostate cancer, colorectal cancer, cervical cancer or malignancy. A further search was conducted by reviewing the references of the index articles for other papers of potential relevance to the aims of the review.

Two independent reviewers then appraised all the retrieved titles on the basis of (i) availability of primary data, (ii) availability of either an abstract or full paper, and (iii) publication in the English language. There were no limitations in terms of study size, study population, the design of the research or the outcomes of the study. However, research articles, reviews, and editorials that were not directly related to the subject of unmet supportive care needs of cancer patients, or were not completely based on primary data were excluded from this review. Also, qualitative studies whose outcome were not specific and could not be quantified were excluded. Data extraction was carried out by the authors using a proforma designed to collect information on the author of the study, year of publication, disease focused on, the location of study, the number of participants, a specific tool used for screening the unmet needs, the main focus of the study and specific outcomes.

## Review

### Measurement of unmet needs

The need to further understand the prevalence, magnitude and significance of unmet needs has led to the development of various measurement and evaluation tools to assess unmet needs in cancers patients. These tools have been developed for a wide variety of subgroups of cancer patients based on the type of cancer, stage of the disease, clinical setting, target audience, or survivors [21]. Although the focus of all these instruments is to assess unmet needs, there is a very wide variation in the parameters of measurement, and especially, the domains of measurement. The domains developed by each of the authors of these tools often have very diverse organizing structures, leading to separations and overlaps across domains [22]. Many of these tools widely apply the Likert-type scale which has been found to be useful in measuring perceived importance, satisfaction, and order of needs as it gives a wide variability of responses and allows patients to express a limited range of subtle responses between extremes [23].

Wen and Gustafson [22] found a total of twenty-four assessment tools which have been found to be useful in assessing unmet needs. However, from this list of twenty-four are fourteen tools that are specific to cancer patients in general (excluding carers and partners, relatives, or stage-specific patients) and these are: Cancer Rehabilitation Evaluation System (CARES), Cancer Rehabilitation Evaluation System-Short Form (CARES-SF), Cancer Patient Need Survey (CPNS), Cancer Patient Need Questionnaire (CPNQ), Supportive Care Needs Survey (SCNS), Home Care Study-Patient Form (HCS-PF), Need Evaluation Questionnaire (NEQ), Patient Needs Assessment Tool (PNAT), Psychosocial Needs Inventory (PNI), Prostate Cancer Needs Assessment (PCNA), Patient Information Need Questionnaire (PINQ), The Derdiarian Informational Needs Assessment (DINA), Information Needs Measure (INM), and the Toronto Informational Needs Questionnaire-Breast Cancer (TINQ-BC). Each of these tools has been examined using specific psychometric evaluation criteria and has been found to have its merits and demerits. The choice of any of these instruments depends on factors such as the strength of its psychometric evaluation criteria, the purpose for which it is to be administered, the domain items it covers, conceptual and measurement model, responsiveness, and the ease of administration (or burden) [21-22].

Out of the fourteen cancer-specific unmet needs assessment tools previously listed, the most commonly used by the studies reviewed was the SCNS, which possesses a relatively high internal consistency (kappa for all the five domains ranges from .87 to .97), strong content validity, easy to understand (fifth grade level), and acceptable to most patients [22]. It comes in two forms: the long form containing 59 items and the short form containing 34 items. Both variants examine unmet needs from five domains: patient care and support, psychological, physical and daily living, health system and information, and sexuality. The items in each variant are spread across these five domains. The short form was a further development of the long form in which weakly correlating items in each domain were consecutively removed until all the remaining items resulted in correlations that were 0.57 or greater [24]. Nonetheless, both forms are fit and recommended for use in assessing unmet needs in cancer patients.

### Magnitude of unmet needs in cancer patients

Across the studies reviewed in this report, many of which were cross-sectional analyses, seven out of the eleven studies reported a significant amount of unmet needs in the health system and information domain, as seen in Table 1 [1,8,10,19,25-27]. Using a range of 1.00–5.00, many of these studies recorded mean values close to or more than the average for the health system and information domain [1,8,10,25-28]. The consistency of this finding across different types of cancers and varying locations in Malaysia, Singapore [29], South Korea [30], Hong Kong [31], Australia [27], Europe, United States [26], Canada [18], and the United Kingdom further establishes the importance of the health system and information domain. Earlier reports had suggested that cancer patients in Asian countries with relatively lower literacy/educational indices had higher information needs when compared with females in Europe or America [32]. However, more recent studies coming from patients in the West have shown that they also have high needs for health information [4,26,33]. Even in studies where needs in the health system and information domain comes second in the order of magnitude of unmet needs, the difference between the first and second (health system and information) on the scale is usually very little. For instance, Aranda, et al. [27] report a mean of 27 for psychological needs domain and 26 for the health system and information domain. Similarly, Sanders, et al. [26] reported mean values of 53.2 and 52.7 for unmet needs in the physical and daily living and psychological needs domains respectively; while a mean value of 52.3 was reported for unmet needs in the health system and information needs the domain. This goes to suggest that many cancer patients are dissatisfied with the information they have been provided with before and during their care.

**Table 1:** Basic characteristics and major findings of studies conducted on unmet needs in patients with cancer

Author [Reference]	Type/number of patients	Tool used	Results	Remarks
Jabbarzadeh, et al. [1]	Mixed cancer; 274 patients	SCNS-LF59	Health system and information (Mean 70.89 SD 16.22) Physical and daily living (Mean 65.92 SD 18.18) Psychological (Mean 59.70 SD 16.80) Patients care and support (Mean 57.71 SD 15.32) Sexuality (Mean 49.39 SD 28.01)	Five of eight of the most frequent unmet needs were in the health system and information domain.
Sanson-Fisher, et al. [3]	Mixed cancer; 888 patients	SCNS-LF59	Fears about the cancer spreading – 40% (Psychological) Fears about the cancer returning – 39% (Psychological) Concerns about the worries of those close to you – 38% (Psychological) To be informed about the things you can do to help yourself get well – 36% (Health system/information) Lack of energy and tiredness – 33% (Physical/daily living)	Of the 10 highest needs items, five were in the psychological needs domain, three items were in the health system & information domain, and two items were in the physical & daily living needs domain
Fong & Cheah [8]	Breast cancer; 101 patients	SCNS-SF34	Health systems and information (Mean 2.48 SD 0.80) Psychological (Mean 2.01 SD 0.53) Patient care and support (Mean 1.93 SD 0.51) Physical and daily living (Mean 1.93 SD 0.62) Sexuality (Mean 1.57 SD 0.65)	
Abdollahzadeh, et al. [10]	Breast cancer; 136 patients	SCNS-SF34	Unmet needs in all domains; 70.7% in health systems and information, 67.8% in physical and daily living, 62.7 % in psychological, 60.5 % in patient care and support and 59.1 % in sexuality	Perceived needs were highest in health systems and information (71%), and physical and daily living (68%) domains
Fitch & Maamoun [18]	Mixed cancers; 115 patients	Supportive Care Screening Tool	Physical unmet needs: fatigue (49%), dry and itchy skin (37%), and sleep difficulties (30%). Emotional: worry (35%)	
Park & Hwang [19]	Breast cancer; 52 patients	SCNS-LF59	About half of the patients reported unmet needs across these three domains: health system and information, patient care and support, and psychological.	Of the top 20 unmet needs, 13 were from health system and information, four from patient care and support, and three from psychological domains
Rahmani, et al.[25]	Mixed cancers; 274 patients	SCNS-LF59	In 18 items of SCNS, more than 50% reported unmet supportive care needs.	Of the 18 most reported unmet needs, eight items were in health system and information domain; five in psychological domain; two in physical and daily living domain
Sanders, et al. [26]	Lung cancer; 109 patients	SCNS-SF34	Physical and daily living (Mean 53.2, SD 51.2) Psychological needs (Mean 52.7, SD 51.0), Health system and informational needs (Mean 52.3, SD 50.9) Patient care support needs (Mean 52.1, SD 50.7)	
Aranda, et al.[27]	Advanced breast cancer; 105 patients	SCNS-LF59	Psychological needs (Mean 27 SD26) Health information needs (Mean 26 SD 28) Physical and daily living needs (Mean 22 SD 22) Patient care and support needs (Mean 9 SD 15) Sexuality needs (Mean 26 SD 27)	
Edib, et al. [28]	Breast cancer; 117 patients	SCNS-SF34	Psychological (Mean 53.31 SD 21.79) Physical needs (Mean 38.16 SD 27.15) Patient care (Mean 37.65 SD 16.45) Health information needs (Mean 31.53 SD 12.17) Sexuality (Mean 27.78 SD 21.91)	
McDowell, et al. [33]	Mixed cancer; 439 patients	SCNS-SF34	Top 5 unmet needs: Lack of energy/tiredness - 26.5% (Physical); Not being able to do the things you used to do - 26.5% (Physical); Fears about the cancer spreading - 24.9% (Psychological); Concerns about the worries of those close to you - 21.8% (Psychological); Uncertainty about the future – 20.6% (Psychological)	The highest ranked unmet supportive care needs were in the physical/daily living and psychological needs domains
*SCNS-SF34 – Supportive Care Needs Survey – Short Form; SCNS-LF59 – Supportive Care Needs Survey – Long Form*				

Unmet needs in the psychological domain also appear to be significant. In a group of 105 patients with advanced breast cancer, Aranda, et al. [27] report that the majority of most prevalent unmet needs came from the psychological domain. In tandem with this, Edib, et al. [28] show that psychological needs were the most prevalent amongst a group of 117 breast cancer patients in Malaysia. It appears that cancer patients in more conservative societies tend to have a large magnitude of unmet psychological needs. Iranian female cancer patients have been reported to have higher levels of stress as a result of cancer diagnosis and its social complications such as divorce, social isolation, and even stigmatization [1,34]. In Malaysia, the majority of unmet needs came from the psychological domain usually as a result of fears about the disease, uncertainties about the future, sadness, thoughts of death and dying, feelings of depression, and worry about the immediate family [28]. This is not to presume that there are no unmet psychological needs in more open western societies. This finding points at the influence of tradition, culture, and societal values on the perception of the disease and a resulting impact on psychological needs.

Even though it can be said that the majority of studies have pointed out the health systems and information domain to be the most important source of unmet needs for cancer patients, the significant number of other studies that have reported otherwise suggests that the pattern of unmet needs may not be strictly static, but that it may be dynamic and constantly change over time. According to Fong and Cheah [8], researchers have shown that shifts in the focus of patients’ perceived needs from health informational needs to psychological needs have occurred over the years as a result of improvements in information delivery. Although this does not hold true as many of the studies which reported health information domain as the most important source of unmet needs have been conducted within the last ten years, it may point to the need to predict these shifts and focus on other likely sources of unmet needs as soon as informational needs are met.

Another factor that may have been responsible for the relatively lower influence of the psychological domain on unmet needs may be the types of communities in which the studies have been conducted. Studies were done in locations with strong community ties and much social support report in which many emotional and psychological needs would have been addressed, within and outside the care facility and report a lower emphasis on psychological needs [8,35]. This is a pointer to the need for cancer patients to have adequate support groups and to also have cancer survivors as members of these support groups [36].

### Factors influencing unmet needs in cancer patients

In comparing results across studies, it is important to take into consideration the peculiarities of each of the study groups. Evidence has shown that sociodemographic and clinical factors influence the pattern of unmet needs perceived and expressed by cancer patients [8,19]. Abdollahzadeh, et al. [10] report that age is the major predictor of unmet supportive care needs as younger patients tend to have more unmet needs. This finding has also been reported by other authors who pointed out that younger females have more needs in the psychological domain [9,13]. Also, breast cancer patients who have children are less likely to have many unmet needs because it has been suggested that the children serve as a source of support and the more the number of children, the greater the social support [10]. Again, patients who are married have been found to have greater unmet needs in the domain of sexuality [9,16,37].

Based on sex, there is no agreement on the influence of specific gender on unmet needs as some studies have reported that females tend to have more unmet needs in the psychological domain, especially if they lived alone [1]. On the other hand, some others have reported that while females may have higher levels of need in specific domains, males have a higher tendency to report a need if they have specific types of cancers such as lung, colon, or rectal cancer [3,38]. To make it more confusing, a few authors have argued that sex is not a predictor of unmet psychological needs [38]. Other sociodemographic factors such as educational attainment [8,39,40], employment status [8], and income level [39] have also been found to influence unmet needs in specific ways.

In the clinical aspect, Park and Hwang [19] show that the mean scores of unmet needs varied between depressed and non-depressed breast cancer patients. Similarly, a closer examination of the rank ordering of the individual items in each of the domains of need in a group of patients with advanced breast cancer shows that the needs of those with advanced disease are different from the needs of the general cancer patient category [27]. Again, tumor sizes are also significant in predicting the presence and magnitude of unmet needs in cancer patients, especially in the health system and information, and psychological domains [39]. In addition, the number of cancer sites has been shown to predict patient needs in the care and support domain, as those who have the disease in multiple sites were found to have higher levels of unmet needs [3].

Unmet needs in the other domains are relevant, as they have been reported in significant proportions in the literature [1,18-19,25-26]. In many studies though, unmet needs in the area of sexuality are the least reported [1,8,10,27-28,41-42]. This may not be a very reliable finding as existing cultural values in many conservative societies forbid and discourage an open discussion of sexuality [28,42]. This may make many patients consider sexuality as low on their list of priorities despite an internal awareness of impaired sexual functioning. Nonetheless, a reduced sexual capacity also has negative effects on the psychological well-being and may contribute to unmet needs in the psychological domain [43-44].

### Implications for practice and future research

One of the major implications of these findings is the desire for assistance that is associated with patients’ recognition of one or more areas of unmet needs [18]. Individuals suffering from cancer are mostly aware of deficiencies in their care and clearly, understand which areas they would like to receive help or assistance. Addressing these deficiencies is therefore very crucial to achieving optimal cancer care and satisfaction. The first aspect of this is the need to develop a concrete and consistent process that regularly monitors patients’ needs with the aid of a reliable, valid, and standardized instrument [45]. The SCNS is one of such tools as it has been proven to objectively and fully assess the major domains of unmet needs of cancer patients. Using these tools place focus on the patient and particularly, the patient’s peculiar concerns and the depth of the desire for assistance. Follow-up assessment can then focus on applying the best interventions that meet the patient’s peculiar needs, in line with the available evidence. It is important to repeat this process throughout the course of treatment, and not just at the commencement of care alone.

In addition to being aware of patients’ unmet needs, it is essential that cancer care providers be knowledgeable about specific interventional pathways that can be used to effectively resolve these unmet needs. A few cancer centers have been able to provide care algorithms focused on patient symptoms and needs using evidence-based practice guidelines which are meant to guide members of the cancer management team in specific clinical situations [18]. Developing an all-encompassing care pathway will require extensive multidisciplinary collaboration with other professionals, as well as home- or community-based organizations, particularly since not all forms of supportive care can be provided at the care center. These guidelines will provide information when the referral is needed, and the pathways for rapid and efficient access to other allied services.

It is important to place the patient at the center of every care efforts and to help them achieve the best possible quality of life despite their cancer diagnosis. As the results of several studies have shown, since one of the major concerns of these patients is in the area of health information, services that address this specific need should be the priority of cancer management. However, active listening, demonstration of empathy, use of open-ended questions and clarification of perceptions about illness concerns are ways to reduce unmet needs in the domain of health information, especially when formal mechanisms to do this are lacking [28]. The use of psychological assessments is also important in order to determine and make interventions about the psychosocial needs of cancer patients.

While it is clear that the magnitude of unmet needs that cancer patients have to deal with is huge, care providers must focus on understanding what these unmet needs are, the possible predictors of these unmet needs and apply evidence-based guidelines to provide care and support for these patients with the aim of treating the illness at optimal quality of life levels. Also, the high prevalence of unmet needs across all the domains points at the need to desperately improve supportive care services for these cancer patients. Services that address informational, psychological, physical and daily living needs should be made priority, especially for those identified to have low social support and are at risk of huge unmet needs.

A closer focus on healthcare providers and support workers on providing and tailoring educational programs will help improve patient-centered care. Considering the significant influence of clinical and sociodemographic factors, more research is required to better understand the specific care needs of cancer patients with much emphasis on their cancer trajectory. In line with this, in-depth research will help to fully understand and provide deeper insight into the influence of specific sociodemographic factors on the magnitude and type of unmet needs perceived. 

## Conclusions

This review has been able to make it clear that cancer patients are vulnerable to a wide range of challenges which result in supportive needs that need to be met. Unfortunately, these needs are not met most of the time, resulting in a reduced quality of life and a general dissatisfaction with care. The results presented by the studies reviewed the placed emphasis on the need to provide solutions to meet patients’ need in the area of health information, psychological care, physical and daily living. At the moment, there is no all-encompassing solution that can help meet all these needs. This then provides an impetus for health professionals to develop and test varying interventions directed towards meeting these unmet needs. This may involve structural changes to the manner of care provision, improvement in the interactional skills of caregivers, improving access to and/or provision of needed resources, and seek for feedback from patients. While it may not be possible to meet all the needs of every cancer patient, as some level of unmet needs may still exist despite interventions. Routine and regular monitoring of unmet needs using the appropriate tools is, therefore, necessary so that cancer care specialists and other health professionals can develop, streamline, and implement specific aspects of cancer care to strategically meet the peculiar needs of their patients.
